# Opportunities and challenges in the development of *Cutaneotrichosporon oleaginosus* ATCC 20509 as a new cell factory for custom tailored microbial oils

**DOI:** 10.1186/s12934-017-0791-9

**Published:** 2017-10-25

**Authors:** Felix Bracharz, Teun Beukhout, Norbert Mehlmer, Thomas Brück

**Affiliations:** 1 Technische Universität München, Division of Industrial Biocatalysis, Lichtenbergstraße 4, 85748 Garching, Germany; 2 Westerdijk Institute, Uppsalalaan 8, 3584 CT Utrecht, The Netherlands

**Keywords:** *Apiotrichum curvatum*, *Trichosporon oleaginosus*, *Cryptococcus curvatus*, *Cutaneotrichosporon oleaginosus*, Oleaginous yeast, Single cell oil, Lipid, Basidiomycete

## Abstract

*Cutaneotrichosporon oleaginosus* ATCC 20509, previously known as *Trichosporon oleaginosus*, *Cryptococcus curvatus*, *Apiotrichum curvatum* or *Candida curvata* D is an oleaginous yeast with several favorable qualities: it is fast growing, accumulates high amounts of lipid and has a very broad substrate spectrum. Its resistance to hydrolysis byproducts and genetic accessibility make it a promising cell factory for custom tailored microbial oils. However, literature about this organism is of varying degree of quality. Moreover, due to numerous changes of the species name, reports are highly scattered and poorly cited. This led to a poor integration of the findings into a unified body of knowledge. Particularly, errors in strain name usage and consequently citation are found even in most recent literature. To simplify future work, this review provides an overview of published studies and main findings regarding the metabolic capacities of *C. oleaginosus*.

## Introduction

### Background

The oleaginous yeast *Cutaneotrichosporon oleaginosus* was originally isolated from factory drains of the Iowa State University Dairy Farm [1]. The organism can metabolize various carbohydrates including lactose and has the ability to accumulate high amounts of intracellular lipids. Although it was first deposited under the name *Candida curvata* D at the American Type Culture Collection (ATCC 20509), it has been published under various names including *Apiotrichum curvatum* [2], *Cryptococcus curvatus* [3], *Trichosporon cutaneum* [4] and *Trichosporon oleaginosus* [5]. Even though, the latter name *T. oleaginosus* has been most frequently used, the diverse designation of the species makes the quantitative acquisition of information difficult. Recently, the review of Yaguchi et al. [6], made an excellent effort to summarize and contrast the data for the prominent oleaginous yeasts *C. oleaginosus and Debaryomyces hansenii*. This review aims to further extend on available data of *C. oleaginosus* to provide the reader a comprehensive but focused overview of the metabolic capacity of this intriguing organism, which most recently has been rendered genetically accessible [7]. More generally, *C. oleaginosus* grows on a variety of complex biomass hydrolysates and even in the presence on fermentation inhibitors. Moreover, it has the ability of accumulate high intracellular concentrations of lipids under specific culture conditions. The cumulative genetic and biochemical features of this organism positions *C. oleaginosus* as a prime candidate to realize ecologically and economically sustainable single cell oil production targeted at generation of biofuels and high value oleo-chemicals.

In a first instance, assembly of available data reports on *C. oleaginosus* requires a delineation of the variable taxonomic classifications for this organism.

Based on a multi-gene sequencing analysis, the phylogeny of the genus *Trichosporon* was recently revised [8]. Together with previous data [9, 10], this comprehensive multi-gene dataset lead to a taxonomic revision of the genus. More recently, a phylogenomic study encompassing genomic information of 17 species also revealed phylogenetic heterogeneity of the genus [11]. Therefore, the previous genus Trichosporon is relocated in the order of *Trichosporonales* now comprising of *Trichosporon* sensu stricto, *Apiotrichum*, *Cutaneotrichosporon*, *Effuseotrichosporon*, *Haglerozyma*, and *Vanrija* respectively [8]. In the course of phylogenetic restructuring, *T. oleaginosus* [12] was placed in the genus *Cutaneotrichosporon*, and renamed in *C. oleaginosus* [13].

The novel genius *Cutaneotrichosporon* actually contains now 13 species and half of them have been found grown either as pathogens or opportunist on humans. The most recent *C. oleaginosus* literature extracted in this review focuses on biotechnological aspects. Another recently published review article mainly compares *C. oleaginosus* with *D. hansenii* [14].

The species found in the genius *Cutaneotrichosporon* do not form basidiocarps, do not show sexual reproduction. Moreover, the fermentation of ethanol is not observed [8]. Apart from its commonly described yeast state, *C. oleaginosus* also grows in filamentous form and produces arthroconidia. In nature, it presumably grows as filamentous fungus in soil and on leaf litter [15]. Oleaginicity appears to be an adaptation to strongly varying nutrient supply, which is supported by the very low maintenance energy of the yeast [2, 16, 17]. Its genome is estimated at 19.8 Mbp, having a high GC content of 61% [18]. In the following section we will elaborate on the available data that governs metabolic capacity, substrate utilization and lipogenesis of *C. oleaginosus.*

## General physiology

### Biochemistry of substrate metabolism

*Cutaneotrichosporon oleaginosus* is able to grow on a variety of carbon and nitrogen sources [6]. However, very little is known about the biochemistry of its metabolic potential. While, cellulase and chitinase enzyme activities have been predicted from *C. oleaginosus* genome annotation [18], the organism does not grow on polysaccharide based materials, such as lignocellulose and chitin [19]. This data suggests, that putative glycosylhydrolase activities are probably of intracellular relevance, i.e. for cell wall remodeling. However, *C. oleaginosus* readily metabolizes a wide range of oligo- and monomeric sugars such as cellobiose, sucrose, lactose and glucose, galactose, galacturonic acid as well as *N*-acetylglycosamine respectively [20–24].

With respect to lactose utilization two lactose hydrolases have been studied [25]. To this end, *C. oleaginosus* harbors a highly active and specific beta-galactosidase that requires metal ions as cofactors. Additionally, a beta-galactosidase activity that does not require metal-ions but has a lower activity and specificity compared to the former enzyme variant has been identified.

While Liang et al. [26] reported arabinose utilization, Meo et al. [27] demonstrated that arabinose is not used for the generation of biomass. These results suggest that *C. oleaginosus* is suffering from arabinose transporter deficiency [28] and/or cofactor imbalance that would enable efficient operation of the arabinose oxidoreductase pathway [29].

In general, xylose is first converted to xylulose 5-phosphate, which in turn can be directed either towards the conventional pentose pathway or the phosphoketolase pathway [30].

Both pathways yield pyruvate as the platform metabolite, which can be further utilized for cellular metabolism. Much like other oleaginous yeasts, *C. oleaginosus* is capable of utilizing glycerol as an efficient carbon source [31] even in the presence of industrial contaminants [32], such as volatile fatty acids (VFA) [33] and ethanol. Most interestingly, *C. oleaginosus* even thrives in the presence of fermentation inhibitors such as 4-hydroxymethylfurfural [26], that is generated during physicochemical pre-treatment processes of complex biomass streams (see “Effects of growth inhibitors in complex biomass hydrolysates” section). Additionally, the organism is able to metabolize simple nitrogen resources such ammonium, nitrate [33] and urea [18, 34], the latter up to a concentration of 1 g/l without growth reduction [32].

### Correlating nutrient preferences and distribution with lipid accumulation

The ability of oleaginous yeasts to generate lipids is highly dependent on the efficacy of carbon source utilization and subsequent application of nutrient stressors other than C-restraints. This section highlights the *C. oleaginosus* metabolic capacity utilize various carbon sources and their influence on lipogenesis.

In general, lipid accumulation can be induced by limitation of specific nutrients. In *R. toruloides*, this was demonstrated for nitrogen, phosphate and sulfur starvation [35–37]. Meo [27] evaluated these limitations for *C. oleaginosus* by employing different C:N, C:P and C:S ratios in two phase fed batch bioreactor cultivations. In the first phase, limitation ratios of batch media were varied. By contrast, in the second phase, limitation ratios of feed were changed. In this bioreactor set-up, C:N ratios of 5–20 g carbon/g nitrogen showed no significant variation. To this end, a maximal lipid content was observed at a C:N ratio of 15 g/g. Nonetheless, subsequent culture feeds indicated that C:N ratios have a significant impact. With decreasing C:N ratio the lipid content decreased moderately. Respectively, a strong decrease between C:N 75 g/g (48% g/g lipids per biomass) and C/N 50 g/g (21% g/g lipid content) was observed. Data is supported by the report of Park et al. [38], whereas Ykema et al. [2] found the critical C:N ratio to be 11 g/g. Variation of C/S or C/P ratios of batch media had little impact on lipid content and no lipid accumulation was induced by sulfate limitation (max 15% g/g lipid content after feeding). Notably, a C:P ratio of 702 g/g was sufficient for the accumulation of 40% g/g lipids, but subsequent feeding required absence of phosphate for intracellular lipids to remain constant.

The reported pH optima for lipid yields differ significantly between pH 4.8 [39] and 7 [33] which fits to a wide spectrum of substrates (natural, semi-defined and synthetic) and fermentation modes applied over various studies. In synthetic media, small differences in pH between 5 and 6, values which are most commonly used for cultivation, have no significant effect on lipid production [27]. Most recently, growth of *C. oleaginosus* was shown to benefit from cultivation in a symbiotic relationship with *Synecococcus elongatus* in a lichen-like structure. This resulted in higher lipid productivity, viability and growth of the oleaginous yeast [40].

There is no comprehensive model capable of predicting biomass yield and lipid content for *C. oleaginosus* grown in arbitrary complex media. Solely relying on carbon source concentration and C:N:S:P ratio for making predictions about lipid content and yield is not sufficient, as interaction effects with other fermentation parameters, such as oxygen supply or absolute cell concentration and concentrations of media constituents, are likely to occur (see “Mechanism and regulation of lipid accumulation” section).

However, when effects of monomeric sugar utilization in the presence of nitrogen limitation was examined, batch bioreactor fermentations [27] demonstrated that the highest biomass and lipid yields can be obtained using mannose as a carbon source, followed by galactose and glucose. By contrast, equivalent experiments with xylose and arabinose resulted in significantly lower biomass and lipid yields, indicating that pentoses are less efficient to sustain growth and lipogenesis. At present, no diauxic effect between hexose sugars has been observed [27, 41]. Most interestingly, supplying a sugar mix rather than individual sugars lead to higher substrate assimilation and maximum growth [27]. However, in the presence of mannose and glucose, galactose utilization was somewhat delayed [27]. Moreover, in the presence of glucose, xylose consumption was significantly decreased [42]. Contrasting the bioreactor data, experiments conducted in shake flasks indicate that glucose, mannose and xylose resulted in comparable intracellular triacylglyceride (TAG) contents. The maximum lipid yield was observed with glucose as substrate followed by mannose and xylose. Again, xylose and galactose resulted in lower biomass and lipid yields. In chemostat experiments with single carbon sources, xylose was the most suitable sugar to achieve a high lipid yield followed by lactose and sucrose [22]. By contrast, Görner et al. found lipid productivity with xylose to be significantly better than with glucose or *N*-acetylglucosamine, both of which were comparable [7]. This suggests a possible involvement of phosphoketolases, which would yield 1.3 mol acetyl-CoA (AcCoa)/100 g xylose as opposed to 1 mol AcCoa/100 g xylose over the pentose phosphate way. Via glycolysis, 1.1 mol AcCoA can be generated from 100 g glucose [30, 43]. In general, metabolic flux from lactose or xylose as carbon source to lipid appears to be less favorable [44]. This may however depend on the presence of further carbon sources and possibly cultivation conditions. The preferred nitrogen sources for lipid accumulation were asparagine and urea, which yielded a higher triglyceride content in *C. oleaginosus* than growth with yeast extract [32]. Most notably, if volatile fatty acids (VFAs) are used as substrate, acetic acid is not only the cheapest option, but also yields higher lipid contents in comparison to butyric acid or propionic acid [45]. Synergistic effects can improve lipid yields. This was shown by Gong et al. [46] using glucose, xylose and acetate in a co-fermentation study. Although, the presence of acetate did not change the capacity of sugar utilization, cell mass and lipid content increased over time and reached 24.5 g/l and 59.3% respectively. The final lipid yield reached 17.5 g/100 g (C:N ratio of 72) [46].

### Effects of growth inhibitors in complex biomass hydrolysates

Acid catalyzed thermochemical pretreatment or saccharification of polymeric biomass substrates, such as cereal straw and wood chips, is accompanied by the generation of fermentation inhibitors [47]. These comprise weak organic acids (acetic acid, levulinic acid), sugar derived furanes (i.e. furfural) and phenolic compounds (i.e. vanillin) originating from lignin breakdown [48]. Compared to other yeast and filamentous fungi, *C. oleaginosus* displays enhanced resistance towards these inhibitors. Therefore it grows comparatively well in a variety of non-detoxified biomass hydrolysates, which represents a significant cost advantage [49]. However, growth is significantly impaired by 1 g/l furfural [49] or 20% w/w final cell dry weight (CDW). However, at higher concentrations CDW remains constant [44]. Inhibition by hydroxymethylfurfural (HMF), *p*-hydroxybenzaldehyde (PHB) and syringaldehyde is low at 1 g/l (< 5% w/w CDW), whereas vanillin at the same concentration reduces CDW by 20% w/w at 1 g/l and 40% w/w at 1.5 g/l. Most notably, the impact of the former inhibitory substances have impact both CDW and the final lipid content (LC) in a similar manner. To that end, furfural reduces LC to 40% w/w compared to the control, while the LC reduction by PHB and syringaldehyde are below 5% w/w. Inhibition of both growth and lipid content can depend on substrate utilization: when *C. oleaginosus* is grown on glucose, in the presence of 1 g/l vanillin a 22% w/w CDW and 10% w/w LC reduction was observed respectively. By comparison, when xylose was the main carbon source, the CDW and LC reductions were 30% w/w CDW and 22% w/w LC [44].

Volatile fatty acids, despite their general suitability as substrate, impair growth at moderate concentrations (43% w/w CDW reduction at 5 g/l for acetic acid) [50]. The inhibitory effect appears to be based on the accumulation of intracellular anions [51]. When using a synthetic broth of VFAs, the threshold for inhibition was found at 6 g/l [45]. However this could be circumvented by raising initial biomass concentration. Up to 40 g/l KAc, only the growth rate and lag phase, but not the final biomass yield are impaired [52]. Interestingly, glycerin concentrations beyond 20 g/l [53] also seem to be inhibitory. Maximum growth rates decrease by 20 and 80% at glycerin concentrations of 100 and 150 g/l respectively [32]. Moreover, an alkaline pH at the start of the fermentation appears to extend the lag phase of the cultivation process [52].

More generally, in the presence of inhibitors, an inoculum of 10% v/v of overnight culture is recommended.

### Cell wall composition

From a technical perspective the chemical composition of the cell wall is crucial if enzymatic methods are to be developed for generation of spheroplasts to establish gene transfer protocols or simply for enzyme mediated cell lysis. Under non-nutrient limiting conditions, the cell wall of *C. oleaginosus* consists mostly of neutral carbohydrates (63% w/w). However, significant concentrations of glucosamine (9% w/w), glucuronic acid (13% w/w) and protein (11% w/w) are also present [15]. Nonetheless, in comparison to other yeast species, such as *S. cerevisiae* (neutral sugar content: 80–90% w/w), the *C. oleaginosus* cell wall displays a relatively low amount of neutral carbohydrates [54, 55]. The mannose content of *C. oleaginosus* cell wall is significantly lower than for *S. cerevisiae* and the high content of uronic acids is unusual for fungi in general. The cell wall is susceptible to digestion by Novozyme 234 [56], which can be exploited for transformation of genetic material.

It is reported that yeasts can accumulate large amounts of disaccharides, trehalose or polysaccharides, such as glycogen or pullulan when metabolically stressed [57]. With increasing nitrogen limitation, lipid content as well as carbohydrate content in *C. oleaginosus* increase [2]. However, lipid accumulation continues even in the stationary phase and is accompanied by a decrease in intracellular carbohydrates [58]. Consequently, carbon source uptake [2] does not appear to be the rate limiting step for the accumulation of lipids. Instead, the subsequent carbon flow to fatty acids (FA) and/or TAG assembly appear to be main bottleneck. This leads to accumulation of sugars in the cell, which act as a “short term” storage product [59]. So far, no qualitatively change in the cell wall sugar profile under nutrient limiting conditions has been reported. However, among the highly upregulated genes under nitrogen limiting conditions is an endoglucanase [18] (Triol1|310356), which is possibly associated with the decomposition of intracellular polysaccharides. Further studies are required to understand changes in the cell wall composition under nutrient limiting and other stress conditions and this data needs to be correlated with lipogenesis under the stress conditions applied.

### The fatty acid profile of the intracellular lipid fraction

The precise fatty acid profile of intracellularly accumulated lipids is essential to determine the technical application of single cell lipids. To that end, Wei et al. [60] reported that *C. oleaginosus* accumulated 88% w/w TAG’s in nitrogen limiting medium, whereas free fatty acids and phospholipids (phosphatidylethanolamine 1.1, phosphatidylcholine 3.5, phosphatidylserine 3.3 and phosphatidylinositol 0.2% w/w) only constituted a minor fraction of total lipid fraction. Dependent on the cultivation conditions, the fatty acid profile of *C. oleaginosus* TAG conventionally resembles that of cocoa butter [60, 61].

In liquid medium, temperature changes between 27 and 33 °C had no significant effect on the *C. oleaginosus* fatty acid (FA) spectrum [62]. By contrast, when grown at 15 °C on solid medium, the *C. oleaginosus* FA content shifts towards longer chain and higher unsaturated fatty acid (FA) content [63]. With regard to cultivation pH, no differences were detected between cells grown at pH 6–7. By contrast, at pH 8 and 9 concentrations of C18:0 and C16:0 as well as C18:2 were increased respectively [52].

A significant change in the organisms TAG profile was detected depending on the carbon and nitrogen source used. In the presence of galactose or arabinose the C18:2 fatty acid pool was enhanced compared to the equivalent cultivations with glucose. However, cells grown on cellobiose, mannose and xylose did not show significant changes in the FA profile compared to glucose as a carbon source [44]. When utilizing complex biomass hydrolysates as growth media, the presence of inhibitory hydrolysis byproducts, such as furfural, PHB, syringaldehyde and vanillin shifted the FA spectrum away from C16:0, C18:0 and C18:1 towards C18:2 [44]. A similar effect was observed when using ammonia as nitrogen source as compared to nitrate [33]. Using VFAs as substrate decreased the amount of C18:2 and use of propionate as substrate made the generation of odd-numbered fatty acids, such as C17:0 or C17:1 possible [33], which are usually not found in yeast. Other methods for the modification of fatty acids have remained unexplored in *C. oleaginosus*: FA desaturase inhibitors were applied to modify the FA spectrum in *Trichosporon cutaneum *and oxygen levels were shown to affect FA spectra in a variety of oleaginous species [64]. Effects of different genetic modifications on FA distribution in *C. oleaginosus* are shown in Table 1.

**Table 1:** Fatty acid mutants of *C. oleaginosus* described in literature

Strain	Verwoert [80]	Ykema [82]		Ykema1 [81]				Hassan [83]		Görner [7]					
	mutant F22	wt	mutant F33	wt	Ufa revertant 22.75	Ufa revertant 25.75	Ufa revertant 26.17	wt	Ufa mutant M3	wt	d9 Elo mutant	d12 Des mutant	LA iso mutant	d9-elo d12-des mutant 1	d9-elo d12-des mutant 2
C16:0	18.7	28.1	25.9	23.7	16	29.9	32.1	34	26	13.5	18.1	14.5	17.6	15	14.9
C18:0	21.5	13.9	20.9	16.6	42.8	26.9	38.4	10.2	36.5	1.7	2.7	2.3	3.9	1.6	2.3
C18:1	40.5	43.7	36.6	11.4	26.8	28.7	17.5	42.8	22	35.7	32.3	42.5	35.1	36.3	42.3
C18:2	8.3	9.6	7.3	7.8	6.6	7.4	6.1	7	8	46.2	27.7	19.7	39.5	11.3	2.2
C18:3				1.4	1.2	1.1	1.4	2.3	4.2	28	1.3	21.1	1.3	17	28.5
C20:2										nd	16.8	nd	nd	9.7	0.9
C20:3										nd	1	nd	nd	8.9	9
CLA										nd	nd	nd	2.6	nd	nd

Values show fatty acid content in % w/w

No value, not measured; nd, not detected; wt, wild type

### Mechanism and regulation of lipid accumulation

Relevance of citrate for lipid biosynthesis has described early as part of the “standard model” of lipid accumulation by Ratledge [57]. While the basic mechanism for lipogenesis under nitrogen limitation has been elucidated in *Y. lipolytica*, there currently is no in-depth regulatory network defining and regulating lipid biosynthesis under nutrient limiting conditions. This applies even more so to the effects of phosphate- or sulfate limitation or the effect of acetic acid.

In this section available biochemical data is summarized, which comprehends molecular mechanisms for lipogenesis under N-limiting growth conditions.

To that end, mitochondrial energy metabolism and management strongly interacts with cytosolic glycolysis reactions. Fatty acid synthesis requires AcCoA and malonyl-CoA (MaCoA). AMP-dependent isocitrate dehydrogenase (IDH) shows activity at very low AMP concentrations, as they are present during nitrogen limitation [65]. In that interaction, citrate is the master metabolite, which regulates intracellular energy (ATP) levels and lipid formation, which ultimately leads to growth stagnation. Citrate accumulates in the mitochondrion and is exported into the cytosol via a citrate/malate antiporter. ATP-citrate-lyase cleaves citrate to acetyl-CoA and oxaloacetate, which [64] is reintroduced into the citric acid cycle [57]. The primary reaction involves ATP-citrate-lyase, which cleaves citrate to acetyl-CoA and oxaloacetate, the latter of which is in turn reintroduced into the citric acid cycle [57]. The presence of ATP citrate lyase (ACL) is considered a defining feature of oil yeasts [66], but non-oleaginous strains with ACL have been described [67]. This suggests, that these organisms may have had the metabolic capacity to form lipids but may have lost this feature during the course of evolution. At present, the supply of NADH has not been fully elucidated, but both introduction of glucose-6-phosphate (by glucose-6-phosphate dehydrogenase, G6PDH) into the pentose phosphate pathway and shunting of pyruvate to oxaloacetate via malic enzyme are likely sources of reducing equivalents to sustain lipogenesis.

Kourist et al. [18] described a transcriptomic analysis of *C. oleaginosus*, comparing nitrogen limited medium containing glucose as carbon source with full complex medium. Amongst the most strongly upregulated genes were amino acid and ammonium transporters. Additionally, many proteases were upregulated to facilitate the recycling of nitrogen in non-essential peptides and proteins. Within the central nitrogen metabolism, equilibrium shifted away from ammonium and glutamine towards glutamate, which in turn is responsible for nitrogen supply to non-essential amino acids over transamination. The mitochondrial isocitrate exporter (Triol1|270035) was not upregulated and hence, the export of citrate to cytosol is possibly not a rate limiting step.

Supply of AcCoA and MaCoA for lipid synthesis was ensured by upregulation of ATP-citrate lyase (ACL) and AcCoA-carboxylase (ACC). These two constituents needed for the production of fatty acids are processed by fatty acid synthases (FAS1, FAS2), which in turn were upregulated as well [18]. NADPH demand for FA synthesis was most likely governed via G6PDH, as glucose-6-phosphate dehydrogenase was upregulated, but malic enzyme (Triol1|326761) was not.

More recently, the role of cell signaling pathways in lipogenesis have been addressed for the first time by Bracharz et al. [68]. To that end, target of rapamycin complexes (TORCs) were identified as central, conserved integrators of stress signals. Involvement of TORC1 in response to nutrient stress was confirmed by rapamycin inhibition. TORC inhibition lead to an enhanced lipid content and a shift in fatty acid spectrum towards a pattern typical for nitrogen limitation. A homology based TORC signaling network assembled by the authors indicates, that cell signaling response to carbon depletion is conserved, whereas response to nitrogen limitation and autophagy are not.

## Process engineering

Optimizing the fermentation process potentially offers the most rapid improvement in biomass yield and single cell lipid production. Therefore, this section elaborates on recently published data with a focus on process engineering in order to increase the productivity.

### Fermentation modes: chemostat, batch and fed batch

Depending on the fermentation mode, substrate and conditions the *C. oleaginosus* biomass and lipid yield vary. As *C. oleaginosus* was isolated from a dairy farm initial reports focused on whey or whey permeate substrate, between 0.13 g/l culture/hour for batch experiment and 0.38 g/l/h for chemostat cultivation could be achieved respectively (see Table 2). Highest lipid productivities were commonly between 0.4 and 0.6 g/l/h, while 1 g/l/h was reported for chemostat cultivation with partial recycling [39] (see Table 3). Fermentations are commonly conducted between pH 5 and 6 (see substrate and growth preferences), while the reaction temperature was almost kept at 30 °C. The average lipid yield over all fermentations was 16 ± 8.4 g/100 g substrate, while the stoichiometric maximum of the lipid yield was 33 g lipids/100 g sugar [69]. Average lipid content was 39.3 ± 14.4% g/g with a maximum of 74% w/w and lipid production was at 14.3 ± 11.8 g/l with a maximum of 49 g/l. Average lipid production was higher at samples quantified by GC/FID (17.1 g/l) in comparison to gravimetric measurements (11.4 g/l), which was however not significant at P = 0.05. Single-stage continuous fermentation requires shorter average residence time than batch fermentation for sugar utilization [70] and showed an average higher lipid productivity. Aeration was commonly adjusted to 1 l/l culture/hour and insufficient supply of oxygen significantly decreased lipid yield and triglyceride content [71].

**Table 2:** Summary of reported batch and fed-batch fermentations of *C. oleaginosus*

Year	Authors	Mode	Substrate	C:N (g/g)	T (°C)	O_2_ (v/v/h)	pH	V (l)	C_L_ (g/l)	Y_LX_ (% g/g)	L_A_	C_X_ (g/l)	Y_LS_ (g/100 g substrate)	Y_LT_ (g/l/h)	C_t_ (h)
1978	Moon [63]	Batch	Whey (lactose)	nd	30	15	5.8	10	9.3	nd	G	nd	20	0.1292	72
		Batch	Whey permeate (lactose)	nd	30	15	5.86	10	15.6	58.2	G	26.8	27	0.2167	72
1978	Moon [1]	Batch	Whey permeate (lactose)	nd	30	1	5.2–5.8	14	9.3	20	F	46.5	15	0.1292	72
1983	Evans [22]	Batch	Semi-defined (glucose)	nd	30	1	5.5	1	33.2	33.2	F	10.2	11.9	0.3689	90
		Batch	Semi-defined (sucrose)	nd	30	1	5.5	1	37.4	37.4	F	11.2	14.8	0.4156	90
		Batch	Semi-defined (lactose)	nd	30	1	5.5	1	39.2	39.2	F	12.5	16.5	0.4356	90
		Batch	Semi-defined (xylose)	nd	30	1	5.5	1	48.6	48.6	F	9.9	17.4	0.54	90
		Batch	Semi-defined (EtOH)	nd	30	1	5.5	1	30.1	30.1	F	8.5	10	0.3344	90
1988	Vega [77]	Fed-batch	Banana juice	nd	30	1	5.2	0.5	6.18	59	G	10.32	nd	0.0858	72
		Batch	Banana juice	nd	30	1	5.2	0.5	7.81	73.7	G	10.6	nd	0.1085	72
		Batch	Banana juice	nd	30	1	5.2	0.5	4.14	28.5	G	14.5	nd	0.0575	72
1988	Ykema [39]	Batch	Whey permeate (lactose)	25	30	> 10%	4.8	1	4.18	18	G	23.2	nd	0.155	27
		Batch	Whey permeate (lactose)	40	30	> 10%	4.8	1	8.85	36	G	24.6	nd	0.199	39
		Batch	Whey permeate (lactose)	70	30	> 10%	4.8	1	11.43	58	G	19.7	nd	0.123	93
		Fed-batch	Whey permeate (lactose)	40	30	> 10%	4.8	1	29.75	35	G	85	nd	0.372	70
1995	Hassan [61]	Fed-batch	Full synthetic (glucose) N-lim	32.4	30	> 20%	5.5	1.8	3.83	22.5	F	17	12.75	0.1472	26
		Fed-batch	Full synthetic (glucose) Fe-lim	3.5	30	> 20%	5.5	1.8	1.62	9	F	18	3	0.0623	26
1996	Meesters [31]	Fed-batch	Semi-defined (glycerol)	20	30	1.2	5.5	0.5	29.5	25	F	118	11	0.59	50
1998	Daniel [93]	Batch	Whey permeate (lactose)	ng	30	ng	5.8	1.5	20	58.8	F	34	20	0.1379	145
1995	Hassan [61]	Batch	Prickly-pear juice	50	30	> 20%	5.5	1.8	8.75	46	G	19.02	21	0.25	35
2010	Liang [94] ⁠	Fed-batch	Crude glycerol		28	0.67	5.5	1.6	13.8	44.2	G	31.2	nd	0.0479	288
2011	Zheng [33] ⁠	Fed-batch	Semi defined (volatile fatty acids)	nd	30	1	7	2	37	nd	G	68.8	nd	0.51	72
2011	Yu [49]	Batch	Wheat straw hydrolysate	nd	28	nd	nd	0.05	5.8	33.5	F	17.2	4.7	0.0345	168
		Batch	Wheat straw hydrolysate, detoxified	nd	28	nd	nd	0.05	4.2	27.1	F	15.6	3.4	0.025	168
2011	Chi [52] ⁠	Batch	Wastewater	10.6	25	nd	66	0.05	0.8	11.1	F	7.5	nd	0.0058	144
2012	Cui [53]	Fed-batch	Semi-defined (glucose)	30	30.2	0.6	5.5	1	17.4	52.9	G	32.9	36	0.0604	288
		Fed-batch	Semi-defined (glucose)	30	30.2	0.6	6	1	21.8	49	G	44.5	26	0.0757	288
2012	Christophe [50]	Fed-batch	Full synthetic (glucose) phase 1	nd	30	0.5	6	4	1.67	16	G	10.46	11	0.0697	24
		Fed-batch	Full synthetic (acetate) phase 2	50	30	0.5	6	4	6.89	51	G	13.5	15	0.1377	50
		Batch	Full synthetic (glucose)	nd	30	nd	nd	0.25	3.31	47.3	G	7	15	0.0345	96
		Batch	Full synthetic (acetate)	nd	30	nd	nd	0.25	0.83	27.8	G	3	19	0.0087	96
2013	Gong [95]	Flask	Corn stover hydrolysate	nd	30	nd	nd	0.05	6	nd	G	nd	nd	0.125	48
		Flask, SSF	Corn stover	nd	30	nd	nd	0.05	7.2	43.4	G	16.5	nd	0.15	48
2014	Xu [76] ⁠	Fed-batch	Mannitol + VFAs	nd	30	0.5	5.5	2	1.3	36.1	G	3.6	nd	0.0271	48
2014	Liang [96]	Flask	Sorghum baghasse hydrolysate	nd	26	nd	nd	0.04	10.8	40	G	27	14	0.075	144
2014	Zhang [75] ⁠	Batch	Acid-thermal pre-treated sludge	18.99	28	nd	6.5	0.15	19.6	52.8	G	37.1	nd	0.4083	48
		Batch	Thermal pre-treated sludge	22.26	28	nd	6.5	0.15	20.4	81.0	G	25.2	nd	0.4250	48
		Batch	Alkaline-thermal pre-treated sludge	15.67	28	nd	6.5	0.15	20.1	51.8	G	38.8	nd	0.4786	42
2015	Beligon [72] ⁠	Batch	Acetate	nd	30	0.1	6	5	0.26	15	G	1.71	6	nd	nd
		Batch	Acetate	nd	30	0.1	7	5	0.4	18.0	G	2.23	9	nd	nd
		Fed-batch	Acetate	10	30	0.1	7	5	12.0	15.0	G	80	5.4	0.2600	60
2016	Gong [46]	Flask	Semi defined (glucose, xylose, acetic acid)	72	30	nd	7	0.05	24.5	59.3	G	14.5	17.5	0.2269	108
		Flask	Corn stover hydrolysate	61	30	nd	7	0.05	11.8	60.8	G	6.9	9.6	0.1229	96
2017	Meo [97]	Fed-batch	Semi-defined (glucose)	15	30	0.1	6.5	0.01	20.4	56.7	F	35.8	11	0.2833	72
		Fed-batch	Semi-defined (galactose)	15	30	0.1	6.5	0.01	18.6	61	F	31	13	0.2583	72
		Fed-batch	Semi-defined (mannose)	15	30	0.1	6.5	0.01	8.9	48	F	18	9	0.1236	72
		Fed-batch	Semi-defined (sugar mix)	15	30	0.1	6.5	0.01	21	58	F	35.5	11	0.2917	72
		Fed-batch	Semi-defined (glucose), P-lim	15	30	0.1	6.5	0.01	11	39.3	F	27.9	5	0.1528	72
		Partial recycling	Semi-defined (glucose)	15	30	2	6.5	5	16.2	52	F	31.5	31	0.4263	38
		Partial recycling	Algae hydrolysate	5	30	2	6.5	5	30.6	53	F	58	43	0.3255	94
2017	Park [45]	Flask	VFA broth with glucose	nd	25	nd	5.5	0.25	3.2	32.2	F	1.0	17	0.0889	36

Cultivation modes include batch, fed-batch, flask, simultaneous saccharification and fermentation (SSF) as well as a membrane bioreactor approach. If not stated differently, lipid accumulation is induced via nitrogen limitation. Aeration is given in volume air per volume culture per hour if not stated differently. In some cases, aeration was adjusted by setting oxygen concentration in the culture over a threshold value (> 10, > 20%). Biomass (C_X_) and lipid concentration (C_L_) are given in g/l, lipid content (Y_LX_) is given in % g/g and total lipid productivity (Y_LT_) is shown as g/l/h. L_A_ shows the method of lipid quantification [fatty acid extraction and GC analysis (F) or total gravimetric lipid analysis (G)]. Substrate yield Y_LS_ is shown in gram lipid per 100 g carbohydrate

Fed-batch fermentations provide for the application of difficult carbon sources which are toxic in higher concentrations. Béligon et al. established a pH regulated feeding strategy utilizing acidic acid as carbon source coupled to the pH. During the cultivation the consumption of acidic acid rises the pH which is compensated by the addition of acidic acid. With this strategy 80 g/l DCW was obtained within 60 h of fermentation containing 18% lipids (g/g). With acidic acid as carbon source a maximum growth rate of 0.26 g/l/h could be achieved [72].

**Table 3:** Overview of reported continuous fermentations of *C. oleaginosus*

Year	Authors	Substrate	C:N (g/g)	T (°C)	O_2_ (v/v/h)	pH	V (l)	C_L_ (g/l)	Y_LX_ (% g/g)	LA	C_X_ (g/l)	Y_LS_ (g/100 g substrate)	Y_L_ (g/l/h)	T_R_ (h)	R_D_ (h)
1983	Evans [22]	Semi-defined (glucose)	nd	30	1	5.5	1	3.94	29	F	45	13.1	0.15	25	0.04
		Semi-defined (sucrose)	nd	30	1	5.5	1	4.54	28	F	53	15.1	0.18	25	0.04
		Semi-defined (lactose)	nd	30	1	5.5	1	5.6	31	F	60	18.6	0.22	25	0.04
		Semi-defined (xylose)	nd	30	1	5.5	1	5.5	37	F	51	18.3	0.28	20	0.05
		Semi-defined (EtOH)	nd	30	1	5.5	1	4	35	F	38	13.3	0.2	20	0.05
1989	Ykema [39]	Whey permeate (lactose)	20	30	> 10%	4.8	1	4.2	20	G	21	nd	0.29	14.3	0.07
		Whey permeate (lactose)	40	30	> 10%	4.8	1	7.2	36	G	20	nd	0.38	18.9	0.053
		Whey permeate (lactose)	40	30	> 10%	4.8	1	30.16	33	G	91.4	nd	1.00	30	0.03
1993	Hassan [98]	Semi-defined (glucose)	38.5	30	> 30%	5.5	1	22	45.6	F	48	22	0.33	20.4	0.049
2009	Zheng [73]	Semi-defined (sucrose)	nd	30	> 50%	5.5	0.5	9.2	15	F	61	7.2	0.33	27.6	0.036

30Cultivation mode is continuous, except for one case of partial recycling. Descriptions of Table 2 apply with the following additions: T_R_ shows residence time in hours and dilution rate R_D_ is given in hours

### Optimization of cultivation on different carbon sources

Statistical methods such as design of experiments (DoE) can be suitable tools for the identification of interacting variables. Moreover, these methodologies enable predictions about fermentation yields. Using a Plackett–Burman design, Zheng et al. [73] tested the influence of different media supplements and cultivation parameters on *C. oleaginosus* CDW with H_2_ producing sludge as substrate. Acetate concentration had the strongest positive impact on CDW, followed by pH, EDTA content and pH. Increasing concentrations of ammonium chloride, magnesium sulfate and peptone had a negative effect. Significant dependent variables were picked for a central composite design (CCD), but as effects of single dependent variables are confounded with higher order effects in the screening, not all significant factors might have been included.

Using the CCD, mainly linear effects and two way interacting variables were identified. Towards that effect, ammonium chloride and acetate concentration were interacting with pH, while the ammonium chloride concentration further interacted with acetate. Additionally, an interaction of the EDTA concentrations with magnesium sulfate was identified. However, as experimental confirmation showed a poor correlation with the predicted CDWs, the model may have to be further refined.

From the CCD, mainly linear effects and two way interactions were found: Effects of ammonium chloride and acetate concentration were interacting with pH, ammonium chloride effect further interacted with acetate, and EDTA with magnesium sulfate. The model validity, however, is questionable, as experimental confirmation fitted poorly with the predicted CDWs.

The abundant availability of carbon rich food waste enables a never dwindling source for fermentative processes. Chi et al. established a combined process using food waste hydrolysate together with waste water for cultivation of different oleaginous yeast and microalgae strains [52]. In this approach, food waste hydrolysate was mixed with municipal waste water and used as fermentation medium. After 6 days of fermentation a biomass of 7.5 g/l was produced containing 28.6% lipids.

Wastewater sludge occurs in large quantities in wastewater treatment plants and is rich in carbon, nitrogen and phosphorous. As renewable material, it is a promising nutrient source for fermentation of oleaginous yeasts [74]. However, a considerable disadvantage is the high nitrogen content preventing high lipid accumulation. By chemical complexation into struvite nitrogen can be removed from aqueous solutions. Unfortunately, Zhang et al. showed that formation of struvite is not sufficient for increasing the carbon to nitrogen ratio and for further increase of oil accumulation [75].

Beside the mentioned domestic waste source, macroalgae algae based biomass has numerous advantages. Macroalgae grows quickly and can be harvested several times per year compared terrestrial crop plants. In addition, on many coast regions macroalgae are accumulated at the beach and has to be removed. In a recent study, Xu et al. used extracts from dried kelp (*Laminaria japocia*) containing mainly mannitol mixed with VFAs as media for fermentation. In this work, a biomass of 3.6 g/l was produced containing up to 48.3% of lipids [76].

Vega et al. optimized CDW of *C. oleaginosus* on banana juice [77] containing 25% w/w sugars. A second order CCD was employed to find optimal pH, concentration of substrate as well as optimal amounts of asparagine and yeast extract supplementation. The factors initial pH (4.8–6.2) and asparagine concentration (15–255 mg/l) were found to be not significant. The factor yeast extract was only significant at juice concentrations under 19% v/v, indicating a lack of nutrients in the juice. Optimum growth was achieved at 21% v/v juice (5% w/w sugars), beyond which growth was impaired. In a two-level full factorial design, the method of sterilization, aeration plug and all previous variables were used as factors. By contrast, significantly higher yields were obtained with filter sterilization over autoclaving, and milk filters over dispo plugs. With a two factor second order CCD, an interaction effect between cultivation temperature and substrate concentrations were found: as juice concentration is increased, the optimal temperatures decreased.

Cui et al. used a Box–Behnken design to estimate effects of substrate (glycerol) concentration, pH and temperature on lipid productivity [53]. Both glycerol concentration (10–30 g/l) and temperature (27–33 °C) had more significant effects on biomass yield than pH (5–6). At pH 6, the temperature optimum was 30 °C and optimal glycerol concentration was 20 g/l. For biomass, the significant factors were glycerol (negative), glycerol quadratic (negative), temperature (negative) and the interaction effect between temperature and pH (positive). For the lipid content, pH (positive effect), glycerol (negative effect) and glycerol (quadratic negative effect) were significant factors. The optimum was at pH 6, 20 g/l glycerol and 30 °C.

In contrast to the former statistical approaches, Ykema et al. [2] applied a kinetic model to predict lipid production in a chemostat culture. Dependent on dilution rate and the C:N ratio, the authors applied a semi-defined medium containing glucose as carbon substrate. The model was capable of predicting lipid production in dependence of C:N ratio, but did not capture the dynamics of the carbohydrate content. Due to its scope, dependence of productivity was modeled only in dependence of few parameters and is therefore applicable only under these defined conditions. The same applies to Browns [70, 78] approach of separating growth into three phases and modeling nitrogen, non-lipid biomass, lactose and lipids using a set of differential equations. The cumulative data indicates, that process simulation are suitable tools to reduce the number of experiment and therefore enable accelerate substrate specific fermentation optimization.

## Genetic modification

Genetic engineering is a route for improving and diversifying single cell lipid production.

To that end, chemical and biological approaches have been employed in *C. oleaginosus*.

While some biological methodologies have proven somewhat successful, the genetic modification of non-conventional yeasts remains challenging. The following section presents a spotlight on current approach to genetic engineering with a focus on *C. oleaginosus*.

### Random mutagenesis and spheroplast transformation

*N*-Methyl-*N*′-nitro-*N*′-nitrosoguanidine (MNNG) and acridine mustard (ICR-170) were suitable mutagens for generating amino acid auxotrophs of *C. oleaginosus*, whereas mutagenesis with ethyl methanesulfonate (EMS) and UV irradiation were less successful [79]. Fatty acid and unsaturated fatty acid auxotrophs were generated by Ykema et al. [79], mutants were created with a modified fatty acid distributions by intraspecific spheroplast fusion with methionine auxotrophs [80] (Table 1). Also revertants were characterized for their modified FA spectrum [81] and growth on whey permeate [82]. Fatty acid mutants were also generated by mutagenesis with EMS [83] and characterized (Table 1). As opposed to the description of Ochsner et al. [56] for strain *Trichosporon dermatis* (DSM70698), plasmid transformation into *C. oleaginosus* did not yield stable transformants (data not published).

### Agrobacterium-mediated transformation

Görner et al. established a method for the stable integration of expression cassettes into the *C. oleaginosus* genome using *Agrobacterium tumefaciens* mediated transformation (ATMT) [7]. Codon optimized yellow fluorescent protein was expressed using the glyceraldehyde-3-phosphate dehydrogenase (GDH) promoter and the respective GDH terminator from *C. oleaginosus*. Selection was done by also expressing hygromycin b phosphotransferase from *E. coli* using a truncated GDH promoter and terminator. Following this proof of concept, different bacterial enzymes for fatty acid modification were expressed to change the fatty acid spectrum of neutral and phospholipids (see Table 1). The approach suffered from the fact that the GDH appeared to be down-regulated under limiting conditions [18], thus limiting productivity of tailor made lipids in *C. oleaginosus*. So far, no other promoters for functional heterologous expression are described.

The reported data suggest, that *C. oleaginosus* is somewhat recalcitrant to genetic modification. *Yarrowia lipolytica* has been described as not favoring homologous recombination over non-homologous end joining [84], which appears to apply even more so to *C. oleaginosus*. Despite the absence of working plasmids for *C. oleaginosus*, there is some evidence of autosomal DNA fragments in the closely related strain *T. cutaneum* (DSM70698) [85]. Due to the absence of homology based methodologies which allow rapid genetic modifications at a specific locus, establishing CRISPR/CAS9 [86] or TALEN mediated [87] genetic transfer systems should be developed. In our hands this endeavor has proven to be a rather complex long term goals, due to the GC-rich nature of the *C. oleaginosus* genome, which complicates design of genetic constructs. Moreover, validation of genetic inserts via sequencing is complicated due to formation of secondary structures when target sequences are amplified by PCR.

## Conclusion

The ability of *C. oleaginosus* to metabolize a broad spectrum monosaccharides and its resistance to fermentation inhibitors designate this organism as a preferred whole-cell biocatalyst able to generate high levels of single cell oils from cost efficient biomass hydrolysates. The fermentation efficacy is enhanced as *C. oleaginosus* is able to simultaneously utilize sugar mixtures or VFAs. The utilization of acetate as carbon source further elevates the intracellular lipid content and circumvents the requirement for nutrient limitation to initiate lipogenesis. The effects of carbon on source lipid content and fatty acid composition is not reported uniformly throughout literature and appears to be interdependent on other fermentation variables, such as media composition and fermentation parameters. The same applies to the fatty acid spectrum, which strongly depends on carbon source, but nitrogen source and aeration also have significant effects.

A process of *C. oleaginosus* fermentation using whey permeate as substrate for production of triglycerides was patented as early as in 1980 [88]. However, since then, no further attempts at commercialization have been conducted. The majority of oil yeast research has been focused on the ascomycetous yeast *Y. lipolytica*, which resulted in a sizable body of literature with over 2400 articles. As the main metabolic paths leading to synthesis of triglycerides are highly conserved, many of the findings could be transferable to other oleaginous yeasts. However, bottlenecks for TAG production differ significantly between *Y. lipolytica* and *C. oleaginosus*: with respect to *C. oleaginosus*, the bottlenecks for lipid production may be manifested prior to TAG assembly. A primary issue may be issues with sugar uptake as described by Tchakouteu et al. [58]. Additionally, our recent data indicates that intracellular free fatty acid concentrations are low and that in contrast to *Y. lipolytica*, DGA overexpression did not provide for an increased TAG content (data not published). With all consideration, the natural lipid content of wild type *Y. lipolytica* (20 [89]–35% w/w [90, 91]) is relatively low compared to *C. oleaginosus*. However, *Y. lipolytica’s* lipid content can be elevated to 45% w/w [90] or even up to 90% w/w with sophisticated genetic engineering [92]. This also yielded in excess of 25 g/l lipids, a value which was achieved by cultivation of *C. oleaginosus* wildtype. Further, *Y. lipolytica* requires engineering for utilization of xylose and sucrose or to overcome strong diauxic effects [92]. Maximum growth rates for *Y. lipolytica* are comparable to *C. oleaginosus* [72]. All of these features are generically included in the wt genome of *C. oleaginosus.* Therefore it can be argued that further exploration of *C. oleaginosus*, despite challenges in its genetic accessibility, is worthwhile. Already as wild type, the strain displays high lipid content, fast growth to high biomass concentrations and a favorable fatty acid spectrum, which has been demonstrated to be modifiable. Further, development of specific genetic engineering tools would provide for development of *C. oleaginosus* as an industrial chassis for sustainable generation of single cell oils. Moreover, systems biology studies may be able to reveal the specific intracellular networks that govern lipid formation in the absence of nutrient limitation. Identification and genetic modification of selective cellular switching mechanisms may be a route to commercial single cell oil production using *C. oleaginosus* as a production host.

## Abbreviations

ACC: AcCoA-carboxylase
AcCoA: acetyl-CoA
MaCoA: malonyl-CoA
ATMT: agrobacterium mediated transformation
ACL: ATP-citrate lyase
CDW: cell dry weight
CO: *Cutaneotrichosporon oleaginosus*
EMS: ethylmethanesulfonate
FA: fatty acid
GDH: glyceraldehyde-3-phosphate dehydrogenase
IDH: isocitrate dehyrogenase
LC: lipid content
MNNG: *N*-methyl-*N*′-nitro-*N*′-nitrosoguanidine
NADPH: nicotinamide adenine dinucleotide phosphate
PHB: *p*-hydroxybenzaldehyde
TAG: triacylglycerides
VFA: volatile fatty acid
